# Community perspective on family medicine and family physician in Saudi Arabia 2020

**DOI:** 10.1186/s12875-021-01604-8

**Published:** 2022-01-21

**Authors:** Manal Abdulaziz Murad, Rawan Maatouk Kheimi, Mohammed Majdi Toras, Rahaf Hussain Alem, Atheer Meshal Aljuaid, Jafar Naji Alobaidan, Hebah Yousef Binishaq, Abdulrahman Ahmed Asiri, Manar Khalid Sagga

**Affiliations:** 1 Department of Family Medicine, Faculty of Medicine, King Abdulaziz University, P.O.Box 80205, Jeddah, 21589 Saudi Arabia; 2 Department of Emergency Medicine, AlNoor Specialist Hospital, Mecca, Saudi Arabia; 3 Department Of Family Medicine, King Fahad Armed Forces Hospital, Jeddah, Saudi Arabia; 4 Faculty of Medicine, King Abdulaziz University, Jeddah, Saudi Arabia; 5 College of Medicine, Taif University, Taif, Saudi Arabia; 6 Alkhaldia Primary Health Care, Ministry of Health, Alhassa, Saudi Arabia; 7 Alrabea Primary Health Care, Ministry of Health, Riyadh, Saudi Arabia; 8 Alnadwa Primary Health Care, Ministry of Health, Riyadh, Saudi Arabia

**Keywords:** Family medicine, Saudi, Perception, Awareness, Attitude

## Abstract

**Background:**

Despite the importance and advantages of family medicine, it has poorly developed in Arab communities when compared to other medical specialties. Therefore, in this study, we aim to investigate the perception of the Saudi population about family medicine and physicians.

**Materials and methods:**

A cross-sectional study was carried out using a self-administered structured online survey tool through the Google Forms platform. The online questionnaire was distributed to all Saudi Arabia’s residents aged more than 15 years. A predesigned questionnaire was used and included items collecting data about participants’ sociodemographic characteristics, awareness/knowledge, and experience/attitudes.

**Results:**

A total of 6974 valid participants were included in the current study, where the age group 25–35 years (37.1%) and 51.7% of them were females. Out of the included participants, 81.3% (*n* = 5671) did not report any chronic illnesses, while the other 18.7% (*n* = 1303) did. The mean awareness and knowledge score for all participants was 9.57 ± 3.39 (out of 20 possible points), while the mean experience and attitude score for all participants was 10.15 ± 2.58 (out of 16 possible points). Patients’ perceptions, whether awareness and knowledge or experience and attitude scores, were significantly correlated (*P*-value < 0.001) to chronic illness status, being a healthcare worker, job, marital status, and gender factors. Moreover, experience and attitude score was additionally correlated to residence region (*P*-value = 0.034) and participants’ nationality (*P*-value< 0.001).

**Conclusion:**

General population in Saudi Arabia were aware about the importance of family physicians and they trust them. The identified predictors should be considered when trying to increase public awareness and enhance the experience with family physicians.

## Introduction

In 1969, family medicine specialty was first inaugurated in the United States by the American Board of Specialties on recommendations of Citizens’ Commission on Medical Education of the American Medical Association, also known as the Millis Commission and Ad Hoc Committee on Education for Family Practice of the Council of Medical Report Education of the American Medical Association, also called the Willard Committee (1966) [1]. The role of family medicine is to take care of various communities regardless of their background or ethnicity. It is meant to provide various health care services that is tailored at enhancing the prognosis of many disorders and improving the quality of life for patients by improving the community’s health. To achieve this, it must depend on firs-contact care, continuous care, coordinated care, and comprehensive care which family medicine is built to provide [1, 2]. Although many benefits have been recorded as a result of family medicine practices, it is rarely researched as it growing to become a vital specialty in the medical field.

Patients’ opinions about primary care services have changed in the past two decades in Saudi Arabia ever since the Ministry of Health (MoH) of Saudi Arabia made it compulsary for admission to hospitals to be through primary care center transfers, except for emergency treatments [3]. This is mostly due to the huge changes in society and patterns of life in many communities which eventually impacts the overall quality of health services [4, 5]. Demands to improve the quality of provided care, the economic burden, and the increased incidence of many morbidities mainly contribute to the organization and enhancement of the provided health care in this field [5]. A successful family medicine practice relies mainly on the relationship between a doctor and his patient. This implies the importance of cooperation between patients and their doctors to provide the required information relevant to their health status [6]. In Saudi Arabia, family medicine practice model is an individual doctor-patient interaction which takes place in primary care centers, which is a governmental public care sector, with a vision to transfer the system into a national health insurance system [7]. It has been reported that patients’ satisfaction with the provided health service is mainly dependant on attending physicians’ practices and attitudes [8]. Moreover, although many advances have been introduced in the healthcare and nursing fields, the association between patients and doctors will always remain the best tool for achieving better prognostic outcomes [9]. A successful doctor-patient relationship is mainly dependant on the satisfaction of the offered healthcare advice and on following these instructions that are provided by the patient’s doctor [10]. Better compliance has been reportedly associated with the enhanced quality of family medicine and the detailed information for patients that consequently lead to more satisfaction and more willingness to cooperate [4, 11]. This indicates the importance of improving family medicine and the relationship between patients and doctors.

Despite the importance and advantages of family medicine, it has been poorly developed in Arab communities when compared to other medical specialties [12]. The specialty of family medicine was first introduced in Saudi Arabia in the early 1980s which was the threshold for many subsequent events that led to big advances in the field [13]. The current family medicine program in Saudi Arabia is run by the Saudi Commission for Health Specialties (SCFHS). Previously the program was divided into a higher diploma of 2 years, and residency of 4 years duration. In 2020, the model of the program was changed to have only a residnecy program of 3 years duration. Previously, only institutes offered residency seats. At the moment, the SCFHS have included many primary care centers across the country to increase the capacity of family medicine residents due to the increasing demand on them and the expansion of more primary care centers across the country. According to the MoH of Saudi Arabia, the total number of primary care centers in 2012 was 2259 [14]. An increase in that number is expected to have hit the 3000 centers by 2021. Nonetheless, there is yet to be any national survey to find the actual number of practicing family physicians in Saudi Arabia and their distribution. Meanwhile, conferences, community activities and research among family medicine physicians is being supervised by the Saudi Society of Family and Community Medicine [15]. However, previously published reports concluded that family medicine services need to be improved in several aspects [16–19]. Additionally, it has been noticed that many patients are not aware of the roles and services provided by the family physician despite the adequate presence of these physicians in healthcare facilities and primary care centers. Not many studies have invistigated the public’s knowledge, experience and satisfaction about family medicine and physicians [20, 21]. Therefore, in this study, we aim to investigate the awareness, attitudes, and satisfaction among Saudi population about family medicine physicians and find the common misconseptions about family medicine in the community.

## Methods

### Study design

This is a cross-sectional study that was carried out using a self-administered structured online survey tool through Google Forms platform from 1st of January 2019 until 30th of December of 2019. The online questionnaire was distributed to all Saudi Arabia’s residents through social media and community online groups with snowballing sampling technique where participants were asked to send the sample for other acquintances from friends or family groups [22]. The inclusion criteria were all residents who agreed to participate in the study and aged more than 15 years. There were no restrictions on gender, nationality, occupation, residence, or socioeconomic level of the participants. The exclusion criteria were all residents less than 15 years, and incomplete data submissions. All methods were performed in accordance with the guidance provided in the Declaration of Helsinki.

### Sampling technique and data collection

Snowball sampling was used to recruit the study participants. An online link to the web-based questionnaire was developed by using Google Forms. On the first section, a Plain Language Information Statement (PLIS) and Consent Form were presented. Only the participants who provided consent and agreed to participate in the study could move to the next section containing the screening questionnaire to confirm the age of > 15 years. The choice of making the cutoff age to be 15 was based on studies that was done stating that adolescents of 14–15 years of age are as competent as adults [23, 24]. Furthermore, in the United Kingdom, those who are 16 years and older can make their own medical decision and provide consent with cases of being as young as 12 years old. Similarly, in Saudi Arabia, the age of which a person can provide medical consent is 16 years old. It is worth mentioning, that in Saudi Arabia, Family Physicians either work in primary care governmental centers or practice in large hospitals. There are yet to be any private GP practice in the country. Upon confirmation, the participants were able to access and fill in the self-administered questionnaire with their personal data being anonymous. An invitation with the online survey link was shared on different social media platforms and online community networks. To avoid potential coercion, healthcare providers were not involved in the recruitment of study participants or collecting data from patients. A total of 6974 valid participants were included in the current study.

### Study instrument

A predesigned questionnaire was used and included data about participants’ sociodemographic characteristics (age, gender, region, occupation, marital status, number of children, educational level, nationality, housing, and monthly income). Regarding face validity of the survey, it was designed by three family medicine physician experts. Afterwards, two public health experts on questionnaire construction methods evaluated it. Regarding language validation, it was performed by translating it from English to Arabic by an official translator and traslated back to English by a different translator. Afterwards, a pilot study was performed to assess the reliability of the survey and was validated using the Cronbach alpha of 0.7 as set point to measure the internal consistency for each question and subdomain. The survey had an explanatory page before the beginning of the survey which explained different terminology such as family physician, general practitioner, internist and surgeons. The questionnaire included items to assess if there is a difference between family physician and general physician, the number of times the participant visited the family physician the present year, participants opinions about the shortage present in the Primary Health Centers (PHCs) if they prefer to visit the emergency department or the PHC, and the actual role of the PHC and the family physician according to the participants’ point of view.

A score was given to the knowledge and awareness or experience and attitude of the participants towards family medicine. Knowledge questions were given a score of [1] for the positive answer and (0) for the negative ones. Every correct answer for diseases treated by the family physician was given a [1] score. Every question was given a score of [1] for the positive answer and (0) for the negative ones. And for the two items: “the role of the family physician is not clearly understood, I don’t see any need for primary health care centers”. Strongly disagree response was given a score of [4], and for strongly agree it was given (0) score. For the question: “opinion about PHCCs”, every negative opinion was given a negative score of (− 1). The highest possible score for knowledge and awareness was 20 and for experience and attitude was 16. A pilot study was conducted to assess the validity and reliability of the developed questionnaire in 10% of the sample size (*n* = 650). Cronbach alpha coefficient to find the reliability for each instrument was utilized. The Alpha coefficient was high for the instruments with a value of 0.83. Following the validation of the questionnaire, we asked all included participants to fill the online questionnaire. The pilot study participants were included in the final study sample when the survey was deemed reliable.

### Statistical analysis

All data were analyzed using R software version 4.0.2 and two-sided *P*-value < 0.05 was considered as statistically significant for all tests. Qualitative data were expressed as numbers and percentages, and the Chi-Square test (χ2) was applied to test the relationship between variables. Quantitiativedata were expressed as mean and standard deviation (Mean ± SD), where Mann-Whitney and Kruskal Wallis Tests were applied for non-parametric variables. In addition, a correlation analysis using the Spearman’s test was done to discover the direction and strength of relationship among the variables..

### Ethical considerations

Data were collected anonymously and no identifying information was attached for this online survey. Therefore, it will not be possible to withdraw from participation, once the completed questionnaire is submitted online. However, the study participants had the freedom to withdraw anytime during the filling up of the questionnaire online. Approval for the study was obtained from the Research Ethics Committee of King Abdulaziz University with IRB approval number [18–20].

## Results

### Sociodemographic characteristics

A total of 6974 valid participants were included in the current study, where the age group ranged from 25 to 35 years was the most common (37.1%) followed by 15 to 24 years (34.6%) and 36 to 50 years (21.9%) groups. The gender distribution was balanced with 51.7% females versus 48.3% males while most of the contributors (62.8%) had the highest education as a bachelor degree. About half of the participants were either single (49.6%) and the other half were married (46.5%) and nearly half of them (55.1%) did not have any children. The monthly income was < 5000 Saudi Riyal in 38.0% of the participants, 9.0% of them were doctors, and 35.5% were health care providers. Saudi nationality was the majority of the patients (95.3%) and 34.7% of them were residing at the central region of Saudi Arabia (Table 1).

**Table 1:** Sociodemographic characteristics of the included participants

Variables		Chronic Illness						*P*-value
		Yes		No		Total		
		n	%	n	%	N	%	
Age	**15–24**	341	26.2	2069	36.5	2410	34.6	< 0.001^b^
	**25–35**	354	27.2	2234	39.4	2588	37.1	
	**36–50**	370	28.4	1154	20.3	1524	21.9	
	**> 50**	238	18.3	214	3.8	452	6.5	
Gender	**Male**	655	50.3	2710	47.8	3365	48.3	0.106
	**Female**	648	49.7	2961	52.2	3609	51.7	
Educational level	**No School**	149	11.5	418	7.4	567	8.2	< 0.001 ^b^
	**Diploma**	204	15.7	760	13.4	964	13.9	
	**Student**	107	8.2	437	7.7	544	7.8	
	**Bachelor**	676	52.1	3695	65.3	4371	62.8	
	**Master**	109	8.4	256	4.5	365	5.2	
	**Doctorate**	53	4.1	93	1.6	146	2.1	
Marital status	**Single**	489	37.5	2973	52.4	3462	49.6	< 0.001 ^b^
	**Married**	727	55.8	2515	44.3	3242	46.5	
	**Widowed**	36	2.8	51	0.9	87	1.2	
	**Divorced**	51	3.9	132	2.3	183	2.6	
Number of children	**No children**	534	41.0	3312	58.4	3846	55.1	< 0.001 ^b^
	**One**	111	8.5	463	8.2	574	8.2	
	**2–3**	190	14.6	901	15.9	1091	15.6	
	**> 3**	468	35.9	995	17.5	1463	21.0	
Home	**My own**	881	67.6	3692	65.1	4573	65.6	0.086
	**Rental**	422	32.4	1979	34.9	2401	34.4	
Income/ month	**< 5000 SR**	396	30.4	2254	39.7	2650	38.0	< 0.001 ^b^
	**5000–10,000 SR**	315	24.2	1343	23.7	1658	23.8	
	**10,000–15,000 SR**	293	22.5	1139	20.1	1432	20.5	
	**15,000–20,000 SR**	173	13.3	550	9.7	723	10.4	
	**> 20,000 SR**	126	9.7	385	6.8	511	7.3	
Job	**Doctor**	98	7.5	528	9.3	626	9.0	< 0.001 ^b^
	**Engineer**	71	5.4	204	3.6	275	3.9	
	**Teacher**	231	17.7	689	12.1	920	13.2	
	**Student**	306	23.5	1840	32.4	2146	30.8	
	**Nurse**	48	3.7	233	4.1	281	4.0	
	**Other**	549	42.1	2177	38.4	2726	39.1	
Are you a health care provider?	**No**	945	72.5	3554	62.7	4499	64.5	< 0.001 ^b^
	**Yes**	358	27.5	2117	37.3	2475	35.5	
Nationality	**Saudi**	1253	96.2	5386	95.0	6639	95.3	0.064
	**Non-Saudi**	49	3.8	282	5.0	331	4.7	
Region	**Northern**	134	10.3	692	12.2	826	11.8	0.003^a^
	**Southern**	230	17.7	1194	21.1	1424	20.4	
	**Eastern**	161	12.4	721	12.7	882	12.6	
	**Western**	278	21.3	1143	20.2	1421	20.4	
	**Central**	500	38.4	1921	33.9	2421	34.7	

^a^ Statistically significant < 0.05; ^b^ Statistically significant < 0.001

### Prevalence of chronic illness

Out of the included participants, 81.3% (*n* = 5671) did not report any chronic illnesses, while the others 18.7% (*n* = 1303) did. Gender, housing, and nationality were all comparable among participants with or without chronic illnesses; however, there were statistically significant differences among those two groups in all other characteristics (Table 1).

**Fig. 1:**
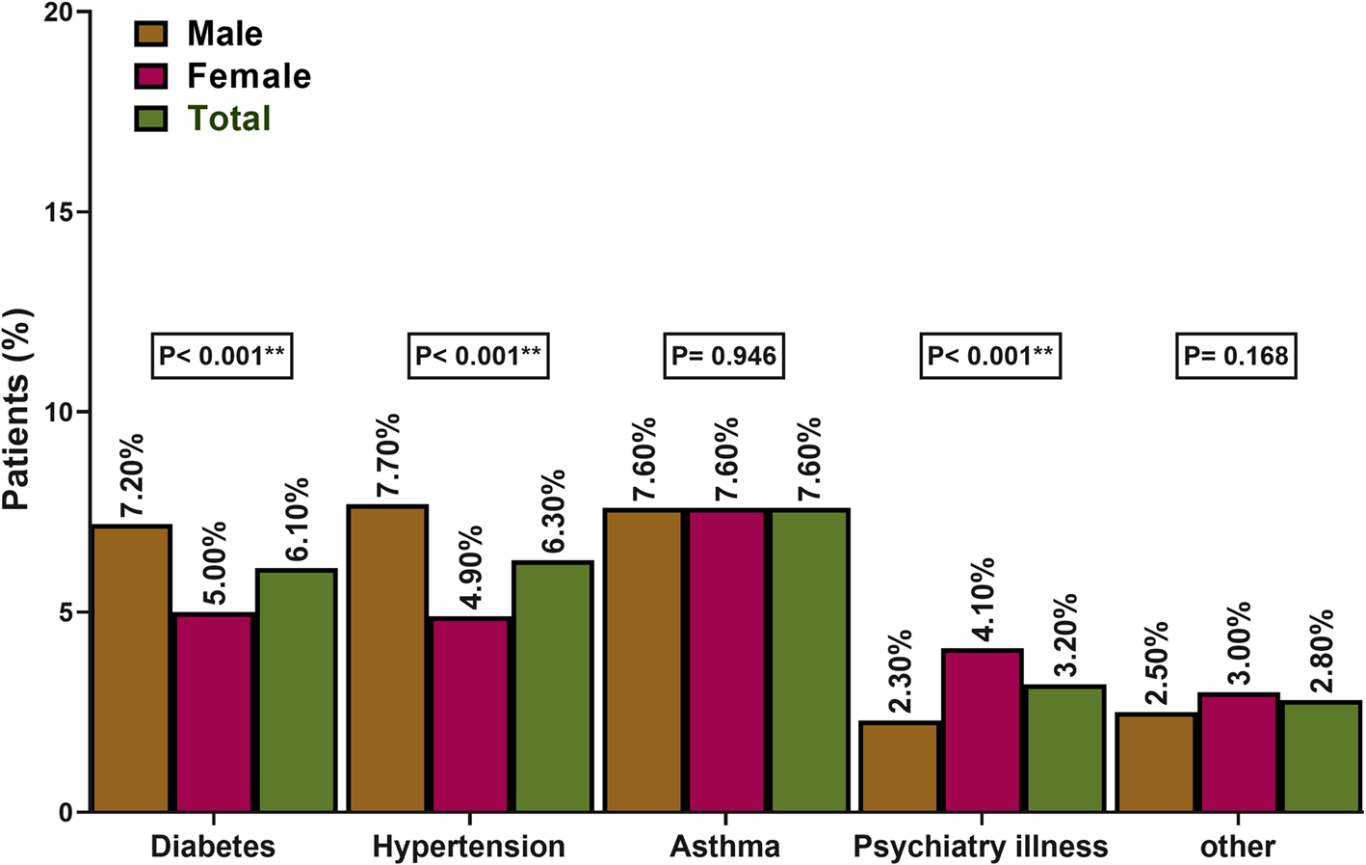
Distribution of different chronic illnesses among included participants

Regarding the distribution of different chronic illnesses, 7.6% had asthma, 6.3% had hypertension, 6.1% had diabetes, 3.2% had psychiatric illness, and 2.8% had other conditions. There was a statically significant differnces (*P*-value < 0.001) among males and females in the rates of hypertension (males: 7.7%; females: 4.9%), diabetes (males: 7.2%; females: 5.0%), and psychiatric illness (males: 2.3%; females: 4.1%) (Fig. 1).

### Perception of family medicine

The mean awareness and knowledge score for all participants was 9.57 ± 3.39 (out of 20 possible points), with a wide range of 1 to 19 and was categorized according to their response to poor knowledge (< 50%), good knowledge (50–75%) and excellent knowledge (> 75%). Out of the included participants, 67.8% of them acknowledged the difference between family physician and general physicians, only 11.4% did not know about PHCs, and 42.7% did not know about the numbers of family physicians per Saudi families. In the same context, only 31.6% were able to identify all listed conditions that family physicians can manage, 52.2% reported that the family physician can manage emergent cases and 93.3% agreed that the physician can also manage simple non-emergent conditions. Interestingly, 56.6% of the participants strongly agreed/agreed that the role of the family physician is not clearly understood (Table 2).

**Table 2:** Awareness and knowledge towards family medicine among the included participants

Variables		Chronic Illness						*P*-value
		Yes		No		Total		
		n	%	n	%	N	%	
Is there difference between family physician and general physician	**No**	219	16.8	824	14.5	1043	15.0	0.035^a^
	**Yes**	846	64.9	3885	68.5	4731	67.8	
	**I don’t know**	238	18.3	962	17.0	1200	17.2	
I don’t know about PHCCs	**No**	1184	90.9	4996	88.1	6180	88.6	0.005 ^a^
	**Yes**	119	9.1	675	11.9	794	11.4	
Family physician can only treat flu and refer you to other specialty	**No**	398	30.5	1805	31.8	2203	31.6	0.013 ^a^
	**Yes**	497	38.1	1924	33.9	2421	34.7	
	**I don’t know**	408	31.3	1942	34.2	2350	33.7	
Number of family physician per Saudi families are	**Enough**	0	0.0	0	0.0	0	0.0	0.004 ^a^
	**Not enough**	793	60.9	3206	56.5	3999	57.3	
	**I don’t know**	510	39.1	2465	43.5	2975	42.7	
Family physician can treat the following								
Chronic diseases (diabetes, hypertension, osteoarthritis, etc.)	**No**	623	47.8	3192	56.3	3815	54.7	< 0.001 ^b^
	**Yes**	680	52.2	2479	43.7	3159	45.3	
Acute disease (flu, gastroenteritis, urinary tract infection, etc.)	**No**	878	67.4	3800	67.0	4678	67.1	0.795
	**Yes**	425	32.6	1871	33.0	2296	32.9	
Gynecological diseases	**No**	1192	91.5	5107	90.1	6299	90.3	0.116
	**Yes**	111	8.5	564	9.9	675	9.7	
Pediatric diseases	**No**	984	75.5	4209	74.2	5193	74.5	0.332
	**Yes**	319	24.5	1462	25.8	1781	25.5	
Dermatological diseases	**No**	1141	87.6	4978	87.8	6119	87.7	0.833
	**Yes**	162	12.4	693	12.2	855	12.3	
Psychiatric illness	**No**	1182	90.7	5080	89.6	6262	89.8	0.222
	**Yes**	121	9.3	591	10.4	712	10.2	
Preventive vaccination	**No**	848	65.1	3573	63.0	4421	63.4	0.161
	**Yes**	455	34.9	2098	37.0	2553	36.6	
All the above	**No**	957	73.4	3810	67.2	4767	68.4	< 0.001 ^b^
	**Yes**	346	26.6	1861	32.8	2207	31.6	
None of the above	**No**	1203	92.3	5220	92.0	6423	92.1	0.737
	**Yes**	100	7.7	451	8.0	551	7.9	
Can family physician manage emergency cases such as cardiac arrest?	**No**	477	49.4	1966	47.5	2443	47.8	0.271
	**Yes**	488	50.6	2176	52.5	2664	52.2	
Can family physician manage non-emergency cases such as simple wounds that don’t require suturing	**No**	100	8.5	323	6.3	423	6.7	0.007 ^a^
	**Yes**	1072	91.5	4784	93.7	5856	93.3	
Do your PHCCs have an urgent care clinic?	**No**	520	57.9	1915	50.9	2435	52.2	< 0.001 ^b^
	**Yes**	378	42.1	1850	49.1	2228	47.8	
	**I don’t know**	0	0.0	0	0.0	0	0.0	
The role of family physician is not clearly understood	**Strongly Agree**	374	28.7	1387	24.5	1761	25.3	0.012 ^a^
	**Agree**	372	28.5	1812	32.0	2184	31.3	
	**Neutral**	334	25.6	1542	27.2	1876	26.9	
	**Disagree**	164	12.6	687	12.1	851	12.2	
	**Strongly Disagree**	59	4.5	243	4.3	302	4.3	

^a^Statistically significant < 0.05; ^b^Statistically significant < 0.001; *PHCC* primary health care center

The mean experience and attitude score for all participants was 10.15 ± 2.58 (out of 16 possible points), with a wide range of 1 to 16. There was no visits of 69.0% of the participants to any family physician during the last year, 10.6% of them reported that they do not trust family physicians, only 52.7% of them identified no problems with their PHCs, and 53.9% showed a preference to visit emergency department over a PHC. Similarly, 11.3% of the participants strongly agreed/agreed that there is no need for PHCs and 30.6% of them just visited PHC to get referrals. However, 69.4% acknowledged how easy and approachable PHC can be (Table 3).

**Table 3:** Experience and attitudes towards family medicine among the included participants

Variables		Chronic Illness						*P*-value
		Yes		No		Total		
		n	%	n	%	N	%	
Number of times you visited your family physician this year	0	723	55.5	4089	72.1	4812	69.0	< 0.001 ^b^
	1–3	402	30.9	1270	22.4	1672	24.0	
	> 4	178	13.7	312	5.5	490	7.0	
Do you trust family physicians	Yes	934	88.2	4068	89.7	5002	89.4	0.146
	No	125	11.8	466	10.3	591	10.6	
Opinion about the PHCCs								
Staff are lacking knowledge	No	1035	79.4	4558	80.4	5593	80.2	0.442
	Yes	268	20.6	1113	19.6	1381	19.8	
Lacking staff	No	962	73.8	4268	75.3	5230	75.0	0.282
	Yes	341	26.2	1403	24.7	1744	25.0	
Difficult to open file	No	1152	88.4	5178	91.3	6330	90.8	0.001 ^a^
	Yes	151	11.6	493	8.7	644	9.2	
Long waiting hours	No	1023	78.5	4439	78.3	5462	78.3	0.852
	Yes	280	21.5	1232	21.7	1512	21.7	
No proper facility (labs/radiology. Etc.)	No	930	71.4	4039	71.2	4969	71.3	0.913
	Yes	373	28.6	1632	28.8	2005	28.7	
Nothing wrong with our PHCCs	No	628	48.2	2673	47.1	3301	47.3	0.489
	Yes	675	51.8	2998	52.9	3673	52.7	
Do you prefer visiting emergency department or PHCC?	Emergency department	693	53.2	3066	54.1	3759	53.9	0.566
	Primary health care center	610	46.8	2605	45.9	3215	46.1	
Don’t see any need for PHCCs	Strongly Agree	82	6.3	266	4.7	348	5.0	0.008 ^a^
	Agree	95	7.3	343	6.0	438	6.3	
	Neutral	266	20.4	1049	18.5	1315	18.9	
	Disagree	386	29.6	1767	31.2	2153	30.9	
	Strongly Disagree	474	36.4	2246	39.6	2720	39.0	
Do you visit PHCC just to get referrals?	No	670	51.4	2859	50.4	3529	50.6	0.012 ^a^
	Yes	424	32.5	1708	30.1	2132	30.6	
	I don’t visit primary health care center	209	16.0	1104	19.5	1313	18.8	
Do you approach easily to PHCC and have easy access to your neighbor center?	No	220	16.9	790	13.9	1010	14.5	0.021 ^a^
	Yes	885	67.9	3957	69.8	4842	69.4	
	I don’t visit primary health care center	198	15.2	924	16.3	1122	16.1	

^a^ Statistically significant < 0.05; ^b^Statistically significant < 0.001; *PHCC* primary health care center

Correlation analyses were performed to test the association of different predictors to patients’ scores. Patients’ perceptions, whether awareness and knowledge or experience and attitude scores, were significantly correlated (*P*-value < 0.001) to chronic illness status, being a healthcare worker, job, marital status, and gender factors. Moreover, experience and attitude score was additionally correlated to residence region (Spearman’s rho = 0.03; *P*-value = 0.034) and participants’ nationality (Spearman’s rho = 0.07; *P*-value< 0.001) (Table 4).

**Table 4:** Correlation between patients’ scores and different predictors

Variables	Awareness and Knowledge Score		Experience and Attitudes Score	
Chronic illness	**Spearman’s rho**	0.04	**Spearman’s rho**	0.09
	**P-value**	< 0.001 ^b^	**P-value**	< 0.001 ^b^
Healthcare worker	**Spearman’s rho**	−0.20	**Spearman’s rho**	−0.04
	**P-value**	< 0.001 ^b^	**P-value**	< 0.001 ^b^
Region	**Spearman’s rho**	< 0.01	**Spearman’s rho**	0.03
	**P-value**	0.900	**P-value**	0.034 ^a^
Nationality	**Spearman’s rho**	− 0.02	**Spearman’s rho**	0.07
	**P-value**	0.065	**P-value**	< 0.001 ^b^
Job	**Spearman’s rho**	−0.08	**Spearman’s rho**	0.07
	**P-value**	< 0.001 ^b^	**P-value**	< 0.001 ^b^
Income	**Spearman’s rho**	0.01	**Spearman’s rho**	− 0.01
	**P-value**	0.266	**P-value**	0.242
Marital status	**Spearman’s rho**	−0.04	**Spearman’s rho**	− 0.07
	**P-value**	< 0.001 ^b^	**P-value**	< 0.001 ^b^
Educational level	**Spearman’s rho**	0.02	**Spearman’s rho**	0
	**P-value**	0.100	**P-value**	0.966
Gender	**Spearman’s rho**	−0.06	**Spearman’s rho**	− 0.04
	**P-value**	< 0.001 ^b^	**P-value**	< 0.001 ^b^
Age group	**Spearman’s rho**	−0.01	**Spearman’s rho**	− 0.01
	**P-value**	0.389	**P-value**	0.470

^a^ Statistically significant < 0.05; ^b^ Statistically significant < 0.001

## Discussion

In this study, we investigated the satisfaction and awareness among the public about family physicians and the factors related to enhancing satisfaction.. This indicates the huge efforts that are being exerted to increase access to universal health care across the country.

We have obtained 6974 results from patients who responded to our questionnaire. According to the demographics analysis, age, educational level, marital status, number of children, income, job, being a healthcare provider, and region were significant variables among the study participants. Family medicine as a specialty involves taking care of many morbidities ranging between simple illnesses to chronic ones as hypertension, diabetes, and asthma regardless of the gender and age of the patients [25]. Although 67.8% of the study participants differentiated between general physicians and family physicians, we found that 56.6% of the included participants did not clearly understand the role of the family physicians. Elagi et al. [26] reported a lower rate of 43.7% among their included participants from Jazan, Saudi Arabia. These results are similar to the previous worldwide reports in Denmark [27], Nairobi [28], and Ireland [29]. Therefore, it has been concluded that patients prefer to seek initial care from specialized personnel of other medical specialties than family medicine physicians [28].

The importance of family medicine was measured by the ability of family physicians to deal with patients and manage their illnesses. In this study, we found a huge variability in what people think family physicians can treat. Almost all participants (93.3%) agreed that family physicians can treat non-emergent cases as simple wounds that do not need suturing or surgical intervention while opinions about whether family physicians can manage emergent caseswas almost the same. However, our analysis showed that most participants trust their family physicians which reflects that a large number of the population believes in their importance. Moreover, around 69.9% of the study population did not agree to this statement “I Don’t see any need for primary health care centers” which indicates the importance of PHCC among the public. Elagi et al. [26] estimated that 67.3% of their study population trusted in their family physicians as the primary healthcare providers. However, the authors reported a rate of 28.3% for patients’ satisfaction. Moreover, Mohamoud et al. [28] reported that only a small proportion of the included participants had confidence in their family physicians’ ability to treat diabetes, tuberculosis, human immunodeficiency virus, anxiety, and depression. On the other hand, previous studies have estimated the rate of satisfaction among the public regarding the roles of family physicians to be ranging between 60 and 90% [21, 30–32]. This indicates that the quality of the offered care by family physicians is hugely variable among the different populations depending on many factors which can hugely affect patient satisfaction.

To identify these factors, we studied the correlation between certain variables and the awareness and knowledge, in addition to the experience and attitudes scores. According to our analysis, having chronic illnesses, being a healthcare worker, job, marital status, and gender significantly affected the awareness and knowledge scores of the included participants. Moreover, the same variables in addition to the region and nationality were also significantly associated with the experience and attitudes of the patients towards family medicine. The significance of different regions and nationalities may reflect that different cultures and circumstances can easily affect patients’ awareness and attitudes. Besides, previous studies have reported that old age and chronic illnesses were significantly associated with seeking and giving the advantage to family physicians [26, 33, 34]. Bawakid et al. [21] also reported that gender was a significant factor affecting patients’ satisfaction. Additionally, the authors have also identified that consulting the same physician was also correlated with patients’ satisfaction. The awareness and attitudes of the public can be improved by enhancing the communication between the family physicians and the public. This can be achieved by providing educational programs to furtherly elucidate the roles of family physicians in addition to further training of family physicians to properly manage the different forms of chronic illnesses and emergencies. Al-Doghaither et al. [31 reported that better communication skills and deep relationships between the patients and physicians were generally associated with better satisfaction and attitudes.

Limitations to our study include the nature of data collection which was online-based with a non-parametric sampling techinque utilized to recruit more respondents, and therefore, sampling bias may have occurred. In addition, some of the survey questions were negatively phrased which makes it susceptible to response bias. Furthermore, using an online-based survey did not allow us to know the response rate of the population nor the denominator. This may have also affected the results in some correlations due to the nature of this sampling. In addition, some variables was not explored sufficiently such as gender and jobs to find which kind were more satisfied.

## Conclusion

The results of this study indicate that the mean awareness and experience scores are generally moderate although most patients trusted family physicians. Having chronic illnesses, being a healthcare worker, job, marital status, and gender significantly affected the awareness and experience scores of the included participants. Therefore, these factors should be considered when trying to increase public awareness and enhance the experience with family physicians by explaining the role of family medicine physicina through campaigns, flyers or public advertisements.

## Data Availability

The datasets generated and/or analysed during the current study are available within the article. However, raw data and the questionnaire due the institute’s IRB polict are available from the corresponding author on request.
